# Fruits of the Earth

**Published:** 1858-05

**Authors:** 


### FRUITS OF THE EARTH.

AMONG the many very useful, because practical books, published by FOWLER & WELLS, New York, is "*The Garden, a Manual of Horticulture: or, How to Cultivate Vegetables, Fruits, and Flowers.*" 166 pp., with engravings; sent in paper for 30 cts., and in cloth 50 cts. The chapter under the heading of "Luxuries of a Fruit Garden," makes the denizen of a city earnestly long for the country. "A friend cultivated," says *The Ohio Farmer*, "a small plot of ground. From a row of currant-bushes, about eight rods long, over two bushels of currants were gathered—the season lasting two months and a half, from June 1. From a bed of strawberry vines, four rods long, and one rod wide, he gathered nearly three bushels of strawberries, the season lasting three weeks from the middle of July. Then the raspberries came on, lasting three weeks. From a number of cherry-trees, he and his friends used a bushel or so, while the children of the neighborhood feasted on them for about two weeks. After the raspberries, the peaches came; and after the first of August, the apples and pears were ripening until winter; while from two or three grape-vines, as many bushels of grapes were gathered and eaten." And yet, there are tens of thousands of farmers in this country, who have whole acres lying waste, year after year, and for want of a little diligence, forego the enjoyment of these delicious things.

